# Supplementary material for: Quality performance and associated factors in Swiss diabetes care – A cross-sectional study
Source: PLoS One. 2020 May 5;15(5):e0232686. doi: 10.1371/journal.pone.0232686 (PMC7200167; doi:10.1371/journal.pone.0232686)
Supplement: S1 Table — BMI = Body mass index, ICPC = International classification of primary care, ATC = anatomical therapeutic chemical code, LDL = low density lipoprotein, HDL = High density lipoprotein, GFR = glomerular filtration rate. (DOCX) [file pone.0232686.s001.docx]

**S1 Table**

| **Disease** | **Identification scheme** |
| --- | --- |
| Obesity | BMI ≥30 (last 12 months)  OR When BMI not available AND ICPC = T82 |
| Hypertension | ATC = 'C02\|C03A\|C03EA01\|C0[78]\|C09[AB]'  OR  ICPC = K85\|K86\|K87  OR two blood pressure measurement ≥ 140/85 |
| Hyperlipidemia | ICPC = T93  OR  ATC = ‘C10A’  OR  Triglyceride >1.7 mmol/l  OR  Total-Cholesterol >5 mmol/l OR  LDL-Cholesterol > 3 mmol/l OR  (sex = 'female' AND HDL-Cholesterol ≤1.2 mmol/l) OR  (sex = 'male' AND HDL-Cholesterol ≤1 mmol/l) |
| Chronic kidney disease stadium ≥ 3b | When last GFR < 45 ml/min and if there is at least one GFR<90 ml/min between date of last measure - 3 month and date of last measure - 24 month |
| Peripheral arterial disease | ICPC = K92 |
| Coronary heart disease | ICPC = K74\|K75\|K76 |
| Chronic heart failure | ICPC = K77 |
| Stroke | ICPC = K90\|K91 |
| Retinopathy | ICPC = F83 |
| Neuropathy | ICPC = N94 |
| Insulin | ATC = 'A10A' |
| Oral | ATC = 'A10B' |
| Antihypertensive | ATC = 'C02\|C03A\|C03EA01\|C0[78]\|C09[AB]' |
| antithrombotic_agents | ATC = 'B01A' AND ATC not = '^B01AD' |
| lipid_lowering | ATC = 'C10' |
