# Supplementary figures and images for: Quality performance and associated factors in Swiss diabetes care – A cross-sectional study
Source: PLoS One. 2020 May 5;15(5):e0232686. doi: 10.1371/journal.pone.0232686 (PMC7200167; doi:10.1371/journal.pone.0232686)

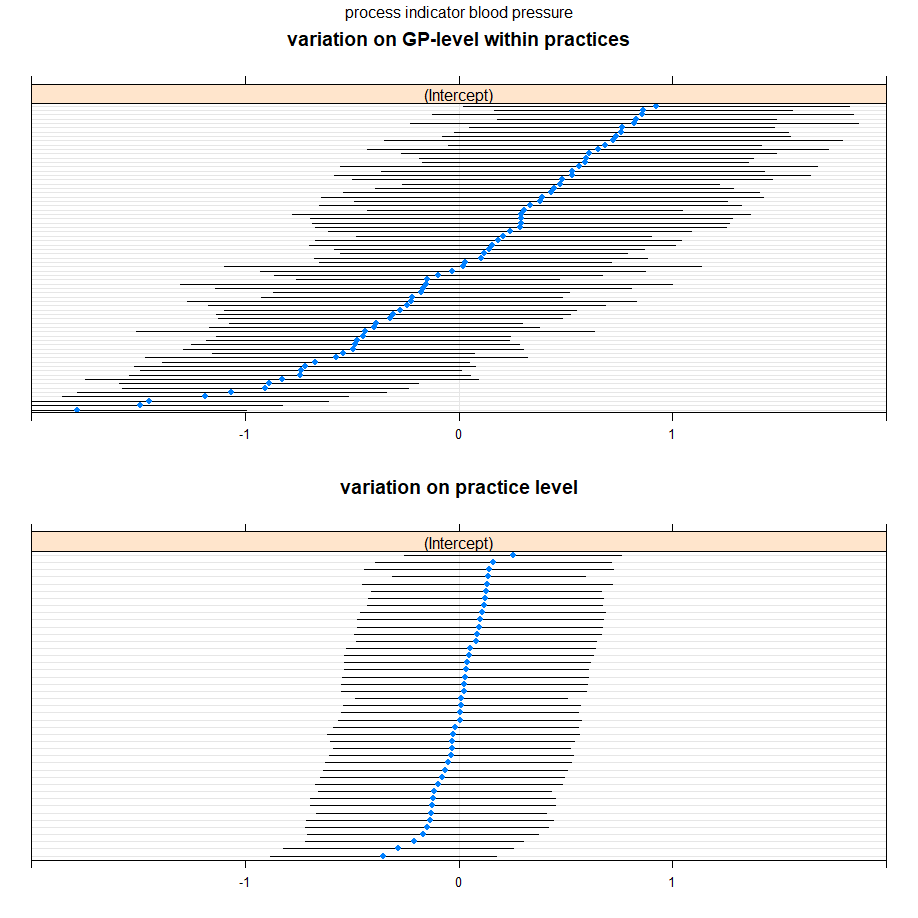

Supplement: S1 Fig — GP = general practitioner. (TIFF) [file pone.0232686.s002.tiff]

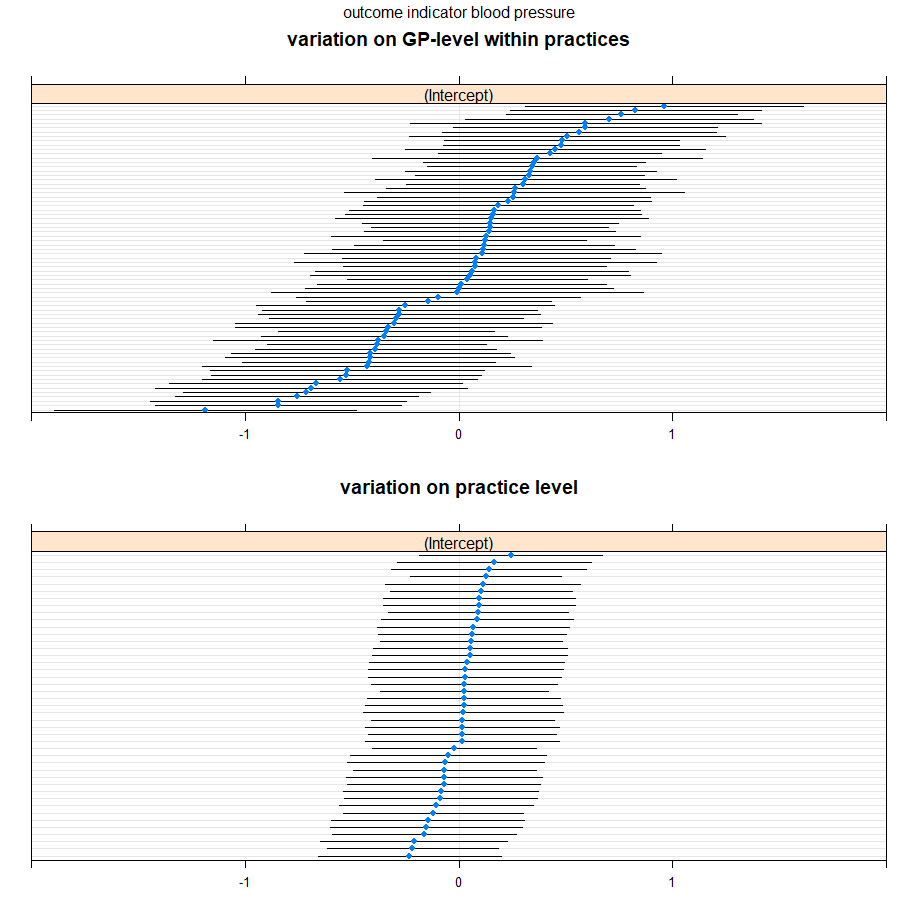

Supplement: S2 Fig — GP = general practitioner. (TIFF) [file pone.0232686.s003.tiff]

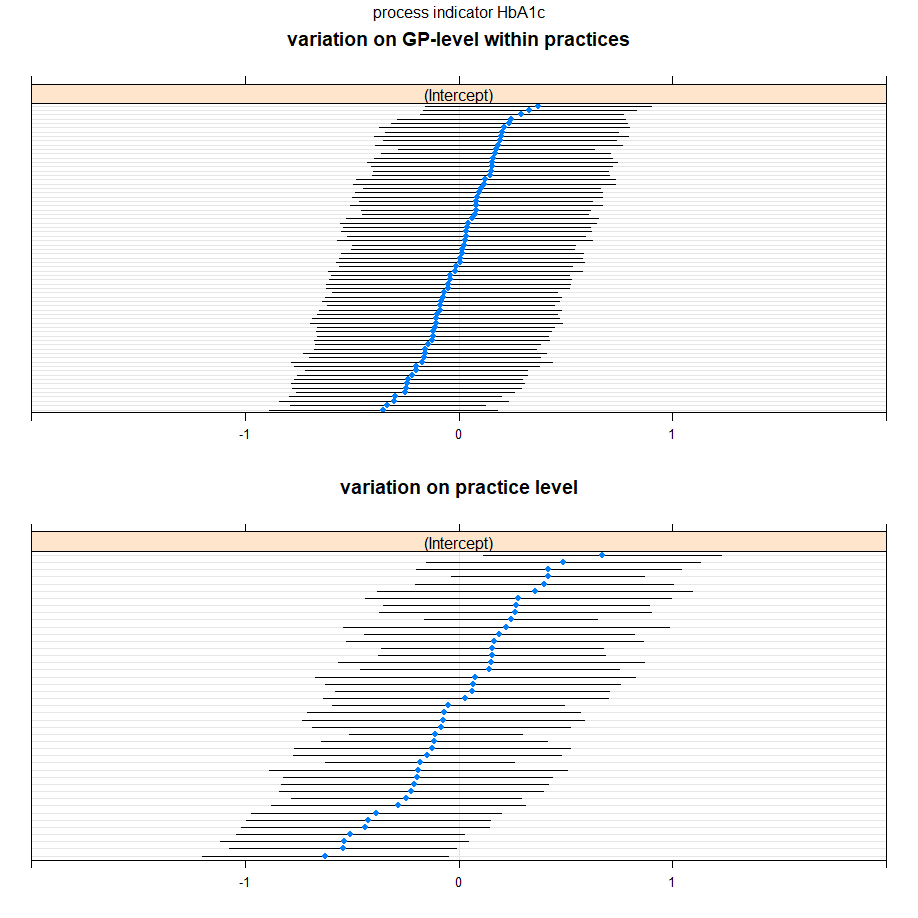

Supplement: S3 Fig — GP = general practitioner. (TIFF) [file pone.0232686.s004.tiff]

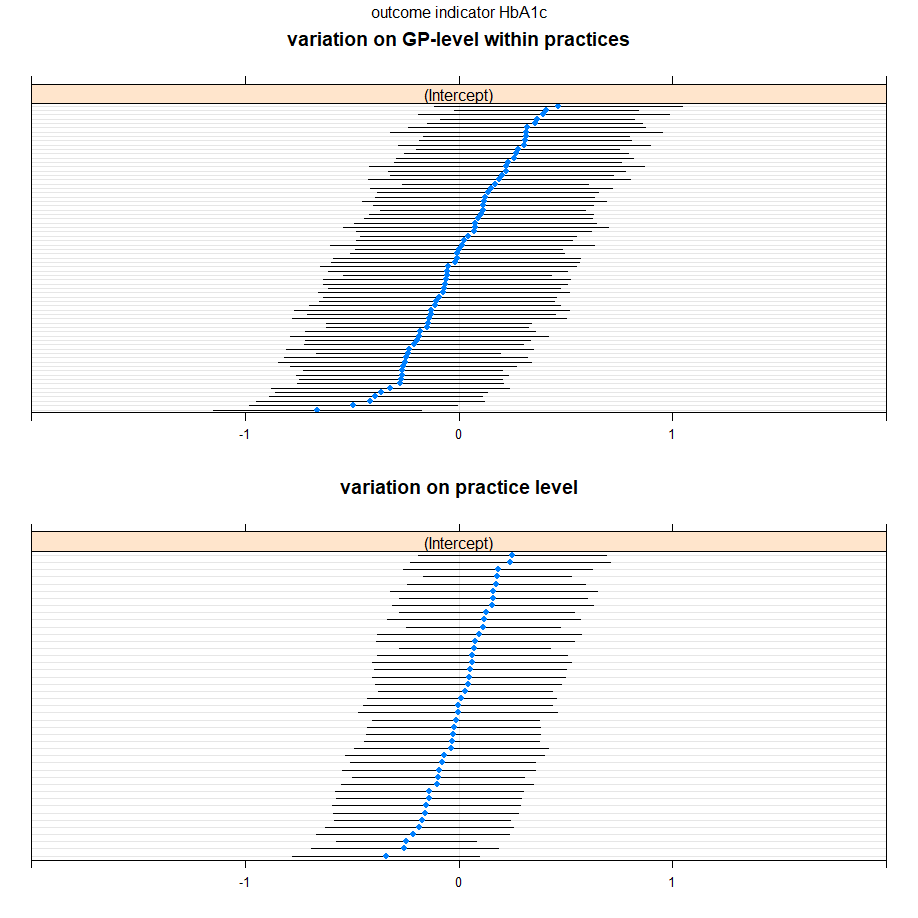

Supplement: S4 Fig — GP = general practitioner. (TIFF) [file pone.0232686.s005.tiff]

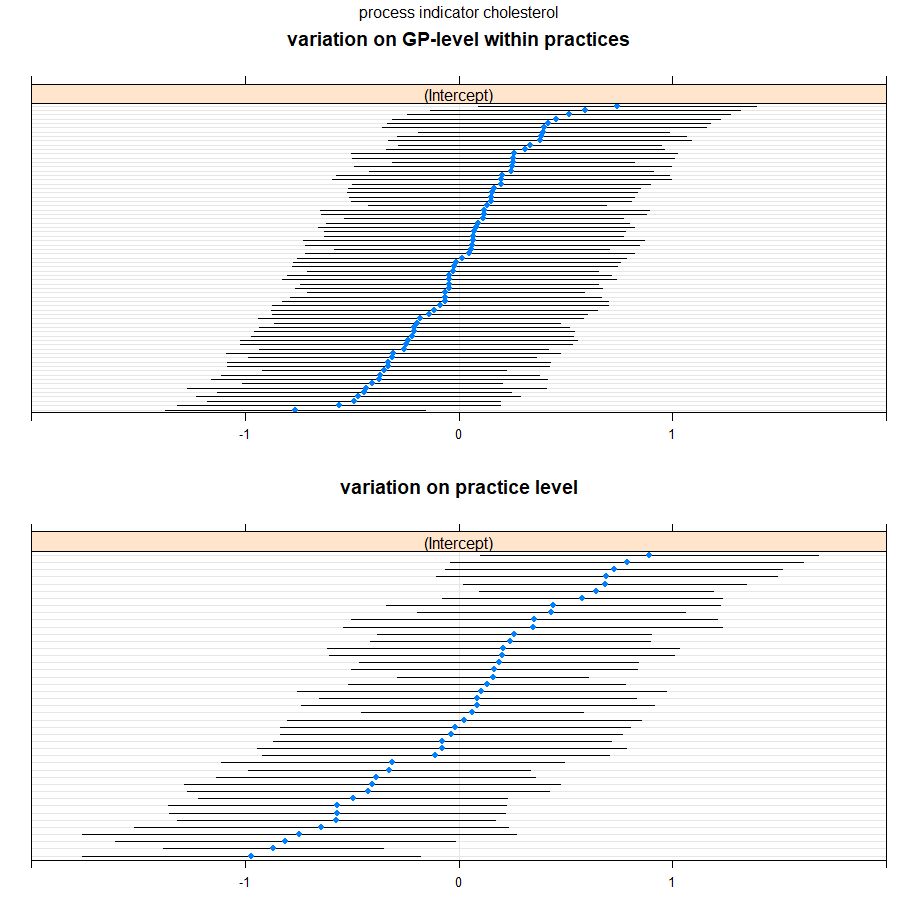

Supplement: S5 Fig — GP = general practitioner. (TIFF) [file pone.0232686.s006.tiff]

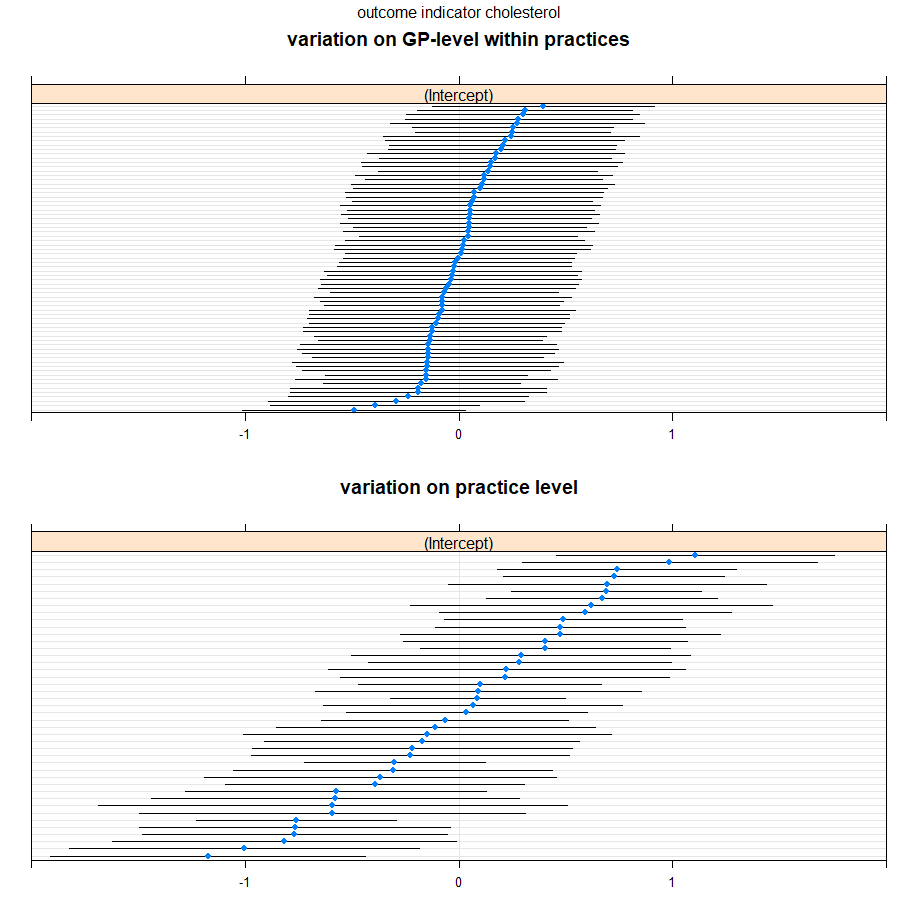

Supplement: S6 Fig — GP = general practitioner. (TIFF) [file pone.0232686.s007.tiff]

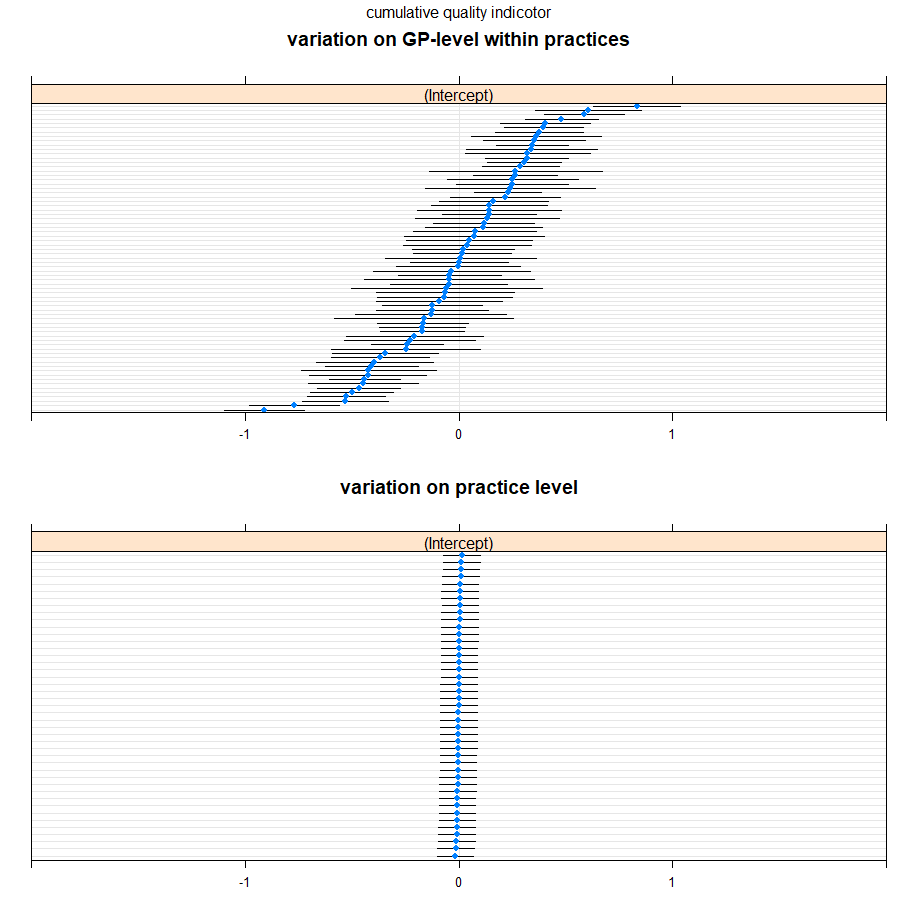

Supplement: S7 Fig — GP = general practitioner. (TIFF) [file pone.0232686.s008.tiff]
