# Supplementary material for: Quality performance and associated factors in Swiss diabetes care – A cross-sectional study
Source: PLoS One. 2020 May 5;15(5):e0232686. doi: 10.1371/journal.pone.0232686 (PMC7200167; doi:10.1371/journal.pone.0232686)
Supplement: Attachment — Submitted filename: Response to Reviewers.docx [file pone.0232686.s011.docx]

| **Reviewer item nr.** | **Reviewer / Editor comment** | **Author response** |
| --- | --- | --- |
| 1.1 | What was the unit of cluster randomization? | Thank you for your comment. The unit of cluster randomization was the practice level. We added this information to the manuscript. |
| 1.2 | What was the difference between the 660000 patients mentioned in this sentence and the 3,833 patients in Figure 1? | Overall, within the FIRE database information on more than 660000 patients was available in August 2019 whereas 3,833 patients were included within the cRCT. For clarification, we have added to the manuscript and the flowchart the according numbers from the time point of recruitment start (June 2018). |
| 1.3 | Please include the definition of “baseline date”. | Thank you for your comment. We included the definition within the text. |
| 1.4 | What was the timing of the retrospectively retrieved patients’ data? How was the timing relative to the timing of baseline? | Thank you for the comment. Information about the comorbidities were collected within the full available patient history whereas laboratory values, vital signs and medication data was collected in the 12 month of baseline assessment. We specified this within the manuscript. |
| 1.5 | (5a) The use of “population” was not clear. What was the unit (e.g., practice, GP, etc.) that the QI performance was defined? (5b) The term “indicator” (or more specifically, QI) was not defined previously. | a) We changed the description within the section "statistical analysis" whereas the level of QI assessment is defined within the last sentences of section "database query and variables".  b) The term QI is defined within the first sentence of the introduction. |
| 1.6 | (6a) To this point, the level of practice was not defined clearly. Was the practice selected first and then GP? (6b) Please define practice type. | Thank you for your comments.  a) We added more information about the inclusion process and defined the practice level more clearly. b) We defined practice type accordingly in the section “database query and variables" |
| 1.7 | (7a) Did “each QI” mean ‘each QI performance’? Please make it clear. (7b) Did the model include each QI performance as a binary outcome? Please make it clear. | Thank you for your comments:  a) We adapted the section and hopefully we made it clear now.  b) We added the relevant information to clarify that the QI performance on patient level was used for the logistic regression (which requires a binary outcome). |
| 1.8 | Please define the cumulative QI performance clearly. | We thank the reviewer for the input. We added the necessary information to the manuscript |
| 1.9 | ‘Sample’ would be a better term than “population”. | Thank you for your input. We changed it |
| 1.1 | What did “the patient population” refer to? | Patient population referred to the GPs patient list size. We changed the wording for better understanding. |
| 1.11 | Please comment on ‘the diabetic status was defined 4 months prior to baseline but QI’s were defined 12 months prior to baseline’. Some patients may not be diabetic when QI’s were collected. | Thank you for this correct remark. We have added these considerations within the limitations section of the manuscript. |
| 1.12 | Throughout this section, I suggest to write non-significant associations as ‘not find evidence of significant associations’ | We thank the reviewer for the input. We adapted the wording. |
| 1.13 | Please provide more details about variations on practice and on GP level in the Methods section. | Thank you for your comment. We added some information in the statistical analysis section saying that we used random effects of hierarchical regression models to assess variance on practice and GP level.  Random effects are a result of using a hierarchical regression model. For interested readers we recommend the following reference: José Pinheiro, Douglas Bates, 2000, Mixed-Effects Models in S and S-PLUS (Statistics and Computing) |
| 1.14 | Please define the “process” and “outcome” indicators in the Methods section. | Thank you for the input. We created a new table listing the indicators. |
| 2.01 | Typo: We assessed the association of individual QI performance of(i.e., process and intermediate outcomes: for glycated hemoglobin, blood pressure and cholesterol) and associations of individual and cumulative QI performance with GP and patient characteristics. | We thank the reviewer for the input. However, we refrained from making the proposed changes, as the meaning would have been changed. |
| 2.02 | “In Switzerland, no data on the pay-for-performance approach exists and the role of QIs has been marginal.” It is unclear here what you mean by the ‘role of QIs’. Do you mean that introducing and monitoring indicators has not led to an improvement in care? | Thank you for your input. We meant that the use of QIs has been marginal. We adapted it accordingly. |
| 2.03 | In general, I would suggest use the term ‘patients with diabetes’ rather than ‘diabetes patients’ where possible throughout the manuscript. | We agree and changed it accordingly throughout the manuscript. |
| 2.04 | number of consultations (OR: 1.020, CI: 1.016-1.023) – keep the number of decimal places used for OR consistent | We thank the reviewer for the input. We changed it accordingly. |
| 2.05 | I appreciate the wordcount here but would be good, if possible, to give the reader some sense of how the different data were collected i.e., at this point when reading about the study I do not know whether the performance indicators are based on extraction from medical records as part of an audit, or another study, and whether the details about the practice and the GPs were collected from a different source | Within the first sentence in abstract/methods we have already specified where the data comes from: "For this cross-sectional study, we used medical routine data from an electronic medical record database." To clarify that the QIs were assessed based on these data we added some information to the abstract. |
| 2.06 | typo: “The use of quality indicators (QI) and contingent incentives aim to improve process and outcomes in clinical practice” | Thank you for pointing out. We changed it. |
| 2.07 | The following paper may also be useful background: • Huang, J., Yin, S., Lin, Y., Jiang, Q., He, Y. and Du, L. (2013), Impact of pay‐for‐performance on management of diabetes: a systematic review. JOURNAL OF EVIDENCE‐BASED MEDICINE, 6: 173-184. doi:10.1111/jebm.12052 | We thank the reviewer for making us aware of the publication. We included the reference. |
| 2.08 | You state that: “Currently, a randomized controlled trial (RCT) testing the P4P approach in Swiss primary care using clinical routine data is ongoing (15). The data of this trial offer the opportunity to study the characteristics and QI performance of the P4P study population and to analyze associations of QI performance with practice, GP and patient covariates.” While I agree this provides justification for the trial, it does not clearly provide justification for the current paper i.e., while it is important to examine the association of different factors (patient, GP, practice level etc) and quality performance. I would suggest including a line here to justify the current study e.g. did it inform the decision to conduct the trial or will it inform the trial and if so, how? I see that in the Methods you mention that data collection for the current study was conducted as part of the trial – it may be worth clarifying this earlier i.e., that the trial was progressing regardless but that it provided an opportunity to explore this association between patient, GP, practice factors and QI. | Thank you for your comment. We clarified in the introduction section that this study is part of the trial and that we used baseline data. |
| 2.09 | Again, relating to study justification, you state “From literature, we know that not only GP, but also patient characteristics influence QI performance (7-10)”. Since the association with practice factors is also mentioned later, should this be introduced earlier? | Thank you for pointing out. We made the necessary changes. |
| 2.1 | Since you mention the introduction of P4P in Switzerland and how the current study uses data from a trial of P4P, I think it would be important to provide some more detail here about the P4P programme e.g. what it involves and when it was introduced. When reading this section it is not immediately clear whether the data you use comes from baseline before P4P was introduced or the control group, or whether you are using all data returned to the database from 2017 to 2019 during which P4P was in place. To help the reader it may be useful to provide a figure or timeline, showing data collection points etc. Figure 1 shows the flowchart and I think looking at this that you used data from the retrospective 12-month period and not the intervention group. If the latter is the P4P group then this suggests your data comes from practices and patients who are not within a P4P system - this should be made clearer. | We thank the reviewer for the comment. We added more information in the methods section to clarify that we used baseline data of the trial. |
| 2.11 | Some acronyms might be unfamiliar to readers (e.g. ICPC, EMR), therefore would suggest spelling these out where possible. | Thank you for pointing out. We changed it accordingly |
| 2.12 | I would clarify what you mean by “Structural data on participating GP are collected at individual project entry.” – does this mean GP/nurses/other staff entered this additional data to the EMR? | We appreciate your comment and clarified it within the manuscript |
| 2.13 | Your use of dates should be consistent - month and years are used in places but “01.01.2017” also used. | We thank the reviewer for pointing out. We changed it accordingly |
| 2.14 | In the ‘Participants’ section you report “71 GPs from 43 different practices were willing to participate”. I feel this should be a result. I see you have reported this information again at the start of the ‘Results’ section. | We appreciate the reviewer’s comment and we reformulated the participants section accordingly. |
| 2.15 | Maybe I missed it, but you do not appear to state whether these are patients with type 1 or type 2 diabetes or both types are included in your sample. On this point, did you consider conducting the analysis separately by type or adjusting for this variable in your regression models? | Thank you for commenting. Unfortunately, due to the patient inclusion criteria we could not accurately separate them into patients with type 1 or type 2 diabetes. We add this to the limitations section. |
| 2.16 | In terms of the statistical analysis, were any further covariates included in the models or just those listed? I would suggest a footnote on the table to clarify these are adjusted OR and what variables they were adjusted for. | Correct, no further covariates were included than those stated in the methods section and reported in table 4. It is our policy to be fully transparent in the methods used and we have no intention to withhold any information on covariates that were included. Furthermore, we would refrain from the comment to the table, as we believe the term adjusted OR is not correct as we examined the independent associations of QI performance with the characteristics. In the table description, we note "Results of hierarchical multivariable regression analysis of quality indicator performance" which in our opinion is precise enough that multiple variables were used and no crude ORs are presented. |
| 2.17 | You should include an explanation of how cumulative QI performance was calculated - do you mean a composite QI here? Was there a scoring system? | We thank the reviewer for the input. We added the necessary information to the manuscript. |
| 2.18 | I know it is mentioned in the introduction, but in connection with the previous comment I would suggest listing which specific QI were used for the current study in the Methods section. You mention the type of data collected but are not explicit about which data corresponds to the QI to examined. | Thank you for the input. We created a new table listing the indicators. |
| 2.19 | You state one objective is “To examine the association of QI performance with practice, GP and patient covariates” (see typo here also), however in the analysis section practice factors are described and listed under ‘GP characteristics’. Therefore, this leads me to think that these factors are being explored at the GP level – I would suggest you keep the terminology consistent throughout the paper, so either describe these as three (patient, GP, practice) or two (patient, GP) separate variable categories or levels and be clear about what comes under each. | Thank you for pointing out. We changed it within the analysis section to be consistent throughout the manuscript. |
| 2.2 | “Number of medications and BMI were not considered as covariates for the regression model as they were used to identify certain comorbidities” | Thank you for the remark. We changed it accordingly. |
| 2.21 | If space allows I think it may be helpful to have a descriptive Table 1 showing the profile of GPs and/or patients. For example, much of the text about the different patient medications and co-morbidities could be captured in a table rather than listing in the text. | Thank you very much for the advice. We created a new table with the patient, treatment and disease characteristics. |
| 2.22 | It is good practice to provide the % and the N – if you have your descriptive table(s) as suggested then you can refer the reader here for the numbers. | Thank you, we did as recommended. |
| 2.23 | typo: “These patients were in 57% male and had a mean age of 68.3 years (SD 13.4).” | Thank you for the remark. We changed it. |
| 2.24 | typo: “On average, patients had eight consultations (IQR: 5-15) at the GPs’ practice (or ‘a GP practice’) in the 12 months preceding baseline.” | Thank you for the remark. We changed it. |
| 2.25 | In the text it is not always clear whether you mean targets achieved or processes performed. These are different and should be made explicit when reporting the results. For example, in line 161 you state “higher age was significantly associated with a higher HbA1c QI performance but with a lower cholesterol QI performance” . Should ‘QI performance’ in this case be taken to mean both measurement of HbA1c (process) and achievement of <7.5% (target achieved)? | As the reviewer has assumed correctly we meant by QI performance the process and outcome indicator when not otherwise specified. As you suggested under item 2.28 we introduced the OR and 95% CI and through this information, we believe that it will be much clearer. |
| 2.26 | In Table 1 you show the % patients achieving QI. There are BP and HbA1c targets mentioned (i.e. <140/85mmHg, <7.5%, < 5 mmol/l). These should have been mentioned in the Methods when explaining the QI to be examined. I realise these are common targets in diabetes management, but I feel it would be important to provide a rationale for using them i.e., are they used in national and/or international guidelines. Later in the manuscript you mention the American Diabetes Association guidelines. If the targets you use are based on these guidelines I would suggest citing them. | Thank you for pointing out. We specified the QIs in the method section and added the according references. The QIs are based on the British Quality and Outcomes framework. For the blood pressure target value, we adapted the target value to the value recommended by the European Society of Cardiology in 2013 for patients with diabetes. |
| 2.27 | (line 146) “93.8% of the patients had at least one comorbidity, whereas 37.9% of the patients had three or more comorbidities.” – do not begin sentences with a numeral. Please amend this in the rest of the manuscript. | Thank you for pointing out. We changed it accordingly. |
| 2.28 | In the section titled ‘Associations with QI performance’ I would report the OR and 95% CI in the text, particularly the significant findings, perhaps referring the reader to the table to review the non-significant findings. | We report now the relevant OR & 95% CI. |
| 2.29 | I suggest rephrasing line 159 “GP characteristics had almost no significant influence on QI performance” as is this is potentially confusing. It would be better to say: ‘Some GP characteristics were associated with QI performance’. I would also advise revising this expression at the start of the discussion. | We thank the reviewer for the input. We adapted the wording. |
| 2.3 | Similarly, I think saying ‘lower’ and ‘higher’ and ‘lowered’ QI performance (lines 158 -166) is potentially confusing as this implies a continuous rather than binary outcome. You are examining the association of these factors with the odds of either performing a process or achieving a target, so I would reword the text throughout this section to make this clear. | Thank you for pointing out. We adapted the section accordingly. |
| 2.31 | (line 168) ‘Cumulative QI’ again is mentioned but it is unclear what is meant here – looking at this table it seems the individual QI are again reported rather than a summary or composite. | We thank the reviewer for the comment. As stated above we have clarified the meaning of cumulative QI performance within the methods section as the number of fulfilled QIs per patient. We are not sure if you refer to the table or figure, but we assume your question will be clarified after reviewing the newly added specifications also concerning the questions/remarks of the other reviewer. The figure 2 represents the OR and CI of the cumulative QI and it makes sense that it looks similar to the individual results. |
| 2.32 | (line 182) “In this study, we explored associations of QI performance with patient, GP and practice characteristics.” – I would rephrase this line, as earlier in the manuscript you lead with the exposure (i.e. GP, practice, patient factors) and finish with the outcome (i.e. QI performance) and this is the convention. In general, I would check this phrasing throughout the manuscript to ensure consistency - I believe the order may be reversed in the results also. | We thank the reviewer for this valuable comment. We adapted the wording throughout the manuscript. |
| 2.33 | I suggest rephrasing this sentence (line 188) “Number of years since diabetes diagnosis was much shorter in our study, which is explicable through limited data availability before GPs participated in the FIRE project.” as it is unclear what you mean – is it the case that people may have been diagnosed earlier but you do not have access to data on these people? | We believe that the reviewer understood the sentence correctly and therefore introduced only minor changes in this sentence. |
| 2.34 | (line 189) “Regarding clinical outcomes, our study population achieved slightly better than the other two Swiss populations.” – I think there may be a word missing here? Slightly better __? | Thank you for the comment. The sentence was omitted. |
| 2.35 | You refer to other studies and how it is difficult to compare their findings your study. I think you need to reflect further on the existing literature and provide a little more detail. For example, you mention (line 193) “Our results for process and outcome indicators for BP and HbA1c were highly comparable to the methodological similar study of van Doorn-Klomberg et al. 2015” - I assume you mean the proportions in terms of targets met or processes performed, but some further explanation would help. | We thank the reviewer for this valuable comment. We adapted the wording. |
| 2.36 | The Stone et al study (line 195) is missing a reference. I think this sentence was meant to be connected to the previous line. | We would like to point out to the reviewer that Stone et al. were cited few sentences above and therefore we did not repeat this citation. However, since this reviewer finds it necessary to again mention the reference, we have again added it within the manuscript. |
| 2.37 | I would not use the phrase “well above 90%” (line 196). Either provide a range or simply state they were over 90%. In general, it is better to give readers a sense of the scale i.e. %. This also applies to the next line where you comment that the other Swiss study found “slightly higher” rates. | Thank you for the comment. We changed it accordingly. |
| 2.38 | (line 200) You state that “Reasons for such poor performances might be clinical inertia to intensify treatment, poor patient adherence or misjudgment by our QI scheme (25, 26).” – I would be interested to know whether you think there is something about the patient or GP population studied which might explain why issues such as inertia would be more common, than, for example, in the Djalali et al. 2014 study which you say is the same EMR database. Does this imply these are similar patients and GPs? | You assume correctly that as the analysis is based on the same database similar GPs and patients are included. Compared to Djalali et al. we included more than twice the number of patients with diabetes. As we have not assessed potential reasons for the poor performance we could only hypothesize about the reasons and clinical inertia is a concept with potential influence on QI performance according to Blonde et al. 2017. We do not believe that it is more common than in other analysis and especially not compared to Djalali et al. 2014, they however, did not comment on this topic. We left the section unchanged within the manuscript. |
| 2.39 | On this point, you mention in the strengths and limitations, using the “full spectrum” (line 236) of patients and that “could show high generalizability of our data due to given patient characteristics.” – I feel the descriptive Table 1 in the Results showing the patient profile would be helpful. In the text you only describe patient in terms of age, sex and diabetes duration, therefore the reader may not understand how this compares to the general population with diabetes. | We thank the reviewer for the comment. We made minor changes to the formulation and introduced the descriptive table 1. We believe therefore that this issue is now understandable for the reader. |
| 2.4 | It is not clear what you mean by ‘misjudgement by our QI scheme’ (line 200) so this needs to be explained. | We made changes to the paragraph and omitted this sentence. |
| 2.41 | You may want to consider what other (unmeasured) factors may influence achievement of QI. For example, is patient socio-economic status something which could have been included in the analysis – I see that you mention this under the limitations section, but you do not expand on why this is relevant to your broader findings. If you feel patient adherence is an issue, then number of medications would have been an interesting variable to examine, though I know this was used to identify comorbidities. The following review may be useful to consult: Hertroijs DFL, Elissen AMJ, Brouwers MCGJ, et al. Relevant patient characteristics for guiding tailored integrated diabetes primary care: a systematic review. Primary Health Care Research & Development. 2018:1-24. | Thank you for addressing this important issue. Unfortunately, this information is not available within the FIRE database and we could therefore not include it within our analysis. We however included these aspects within the discussion section as you suggested. |
| 2.42 | You might consider commenting further on the finding that increased patient age and longer duration were both associated with poorer performance on HbA1c QI, while not associated with BP and cholesterol. For example, what are the implications? Is this something other studies have found? Perhaps this could be linked with the limitation you mention regarding having approximated duration by using the first record as the onset of diabetes. | We thank the reviewer for the comment. We like to point out that patient age is not associated with poorer performance regarding HbA1c but better performance whereas longer disease duration was only associated with a poorer fulfilment of the clinical QI for HbA1c but not for the process QI. It makes clinical sense and it is recommended to treat patients with longer disease duration less strict regarding clinical targets of HbA1c, which we stated in the manuscript. We could detect this negative association only for the clinical QI for HbA1c whereas the fulfilment of the clinical QI for BP was not associated with disease duration and the fulfilment of the clinical QI for cholesterol was positively associated with disease duration. In our view, these findings do not allow to link it consistently to the limitation regarding approximated disease duration. It is, however, addressed within the limitations. |
| 2.43 | (line 205) typo: “Regression analysis revealed that included characteristics had no to only very small effect on process and outcome indicators.” | Thank you for pointing out. We made some changes to this sentence. |
| 2.44 | (line 206) What is meant here?: “Most significant effects were found on patient level, and the largest effect was an increasing number of diabetes-relevant comorbidities.” The largest effect on what? What this a positive effect? By ‘most significant effects’ do you mean that more patient level factors were associated with QI performance? | Thank you for your remark. We introduced changes to the sentence to make it clear and more accurate. |
| 2.45 | Lustman et al. 2016 is missing a citation | Please consider the answer to item 2.36. |
| 2.46 | (line 224) “Which is contradictory ing to Lustman et al. 2016 reporting an effect of continuity of care on the major clinical outcomes.” What is meant here? They found continuity of care had a negative effect on outcomes? How did they define continuity of care in their study? | Thank you for pointing out. We changed it |
| 2.47 | (line 227) typo: “However, our model also revealed that unexplained variation exists between practices and GPs, which cannot be explained by our model” | Thank you for pointing out. We changed it |
| 2.48 | Where you mention other unmeasured GP factors such as culture, working style etc., it may be worth citing some qualitative work which has been done in the area of barriers and facilitators of diabetes management in primary care. Some examples below: • Rushforth B, McCrorie C, Glidewell L, et al. Barriers to effective management of type 2 diabetes in primary care: qualitative systematic review. Br J Gen Pract. 2016;66(643):e114-27. • Zhang JQ, Van Leuven KA, Neidlinger SH. (2012) System Barriers Associated With Diabetes Management in Primary Care. The Journal for Nurse Practitioners 8(10):822-7. • Nam S, Chesla C, Stotts NA, et al. (2011) Barriers to diabetes management: patient and provider factors. Diabetes Res Clin Pract 93(1):1-9. | We thank the reviewer for pointing us at the relevant references. We found them very helpful and introduced them in our manuscript as appropriate. |
| 2.49 | I would advise not using the term ‘diabetic patients’; instead ‘people with diabetes’ or ‘patients with diabetes’. See https://care.diabetesjournals.org/content/40/12/1790 and the discourse around the use of terminology. | Thank you for your valuable input we changed it throughout the manuscript. |
| 2.5 | In the strengths and limits you mention ‘potential missing data’ (line 244). It would be helpful report in the Results whether any of the variables you used had missing data. As you only report % in the text it is difficult for the reader to determine the level of missingness. | Thank you for your comment. As we point out in the limitation section, we cannot distinguish between real missing data (not measured) and technical missing data (not available for FIRE). This has implications on the QI performance as we had to assume that missing data is real missing data. Therefore stating % of missingness in the descriptive table is not valuable. The maximum possible percentage of missing data is represented in the proportions not fulfilling the process indicators. Those not fulfilling the indicator had no record of the according measure but unfortunately, we cannot distinguish between real missingness and technical missingness. We clarified this issue in the limitations section. |
| 2.51 | The meaning of this sentence (line 250) is unclear and needs to be rewritten “This bias is probably of larger issue with lower performance rates as in QI for cholesterol.” | This sentence was eliminated due to restructuring of the paragraph. |
| 2.52 | (line 252) You mention “We tried to minimize the amount of missing data by setting a minimum standard of data availability for each GP to be contacted for recruitment.” . This is important and should be mentioned earlier in the Methods section when explaining participants. From the way you describe it in results “125 GPs met the data availability criteria of a) delivering data on a regular basis since 01.01.2017” it was not clear to me that there was a threshold, or what is meant by ‘regular basis’ – do you mean they had to deliver a complete dataset annually or at some other interval? | We thank the reviewer for the input. We refined the three points that GPs had to fulfil in the methods section. If those were not fulfilled, we did not contact them regarding the participation in the trial at all. |
| 2.53 | I think there is potential to expand on your overall implications and conclusions and make these stronger and better situate them in the broader literature. For example, given there were a number of covariates which you could not explore in your analysis: 1) do you feel there is scope for future work (and what would this look like?), 2) is there scope to expand on (e.g. to include further variables) or improve (e.g. your comment about missingness) the type of data collected in the FIRE database, 3) do you think this database should or can be used for monitoring quality of care and providing feedback to GPs/practices in the future? | Thank you very much for the comment. We expanded on the implications and conclusion and addressed your recommendations. |
| 2.54 | As I commented earlier there needs to be a more explicit justification for the current study in the Introduction. Once this is made clearer it will be easier for you to link your conclusions and implications back to the reason for conducting the study. For example, considering your conclusion regarding the ‘room for improvement’, was your initial aim to determine whether the quality of Swiss diabetes management needs to be improved and how to do so and/or identify factors to inform case-mix adjustments? How do your findings inform the P4P trial? Earlier in the Introduction you mention the lack of understanding around QI performance within P4P systems, particularly in Switzerland. However, in the Discussion there is no mention of P4P and how your findings relate to research from other countries which have introduced P4P – for example, the Quality and Outcomes Framework in the UK. I understand this omission if you used data from the patients and GPs who were not in the P4P intervention group. However, even if you looked at QI performance in a group who are not within P4P system, still I think you need to be clearer about the purpose and relevance of this current study, how it relates to the trial and how it fits in with the bigger picture. | We thank you for your comments.  1. We added more information on the explicit justification for the study within the introduction as mentioned above and that the data used was assessed before the P4P scheme was introduced.  2. We believe that it is essential to check performance measures at baseline and to investigate factors associated to potentially include them in the assessment. However, we found only limited associations between the investigated characteristics and the QI performance, indicating that some meaningful characteristics might be missing. The implications of the study are now better addressed in the conclusion. |
| 3.1 | The aim of this study was to describe QI performance levels and to analyze associations of QI performance with practice, GP and patient covariates. The authors are to be commended that their research is timely. Diabetes prevalence is rising and the condition increases the risk of morbidity and mortality. | We thank the reviewer for considering the importance and timeliness of our manuscript. |
| 3.2 | It might strengthen the manuscript if the Introduction section were rewritten to link the P4P approach better to the methods and results section and the variables measured and analysed in the current study. It is not clear how financial incentives were taken into account in the Result and Discussion sections. | We thank the reviewer for this comment. The other reviewers also pointed at this issue and by implementing the comments the manuscript improved and the introduction section reconciles now with the other sections. |
| 3.3 | Design and methods. Data regarding participating GPs and the care of their patients are appropriate in relation to the aim of this study. Nevertheless, the GP’s are a rather selected group compared to the total population of GP’s in primary care. The authors may consider to further illuminate potential bias related to selection of participants. As to patient data, there is a lack of information related to clinical variables, ex. diabetes type etc. | Thank you for pointing out these factors. We expanded the section on the representativeness of GPs and the potential biases of GPs participating within our study. As the reviewer also pointed at the missing information about the type of diabetes: due to the patient identification scheme, we are unfortunately not able to distinguish between type 1 and type 2 diabetes. We address this issue now within the limitations. |
| 3.4 | Results. The data support the conclusion. However, the interpretation of the results should be discussed with more caution. Limitations of the study are not sufficiently outlined. | We thank the reviewer for the comment. By addressing the many comments from the other reviewers, we could improve our manuscript and results are now presented more carefully and improve our discussion and limitation section. |
| 3.5 | The discussion section is interesting. However, a wider perspective in the discussion section might benefit the manuscript. If the literature references could include more studies from other countries in addition to Swiss primary care, it might be a more interesting paper also for international readers. | We added more information from other European countries. However we think the comparison with other Swiss studies is of particular interest as the role of QI is only emerging and the Swiss health care system is somewhat different from other health care systems, hence comparability with Swiss study population is essential for interpretation of our data. |
| 3.6 | In conclusion, this is an interesting and timely manuscript. More effort might however, be put in further clarification about the sample population, and how selection bias might have affected the results | We thank the reviewer for the valuable comments and hope to have addressed all your concerns. |
