# Supplementary material for: Quality performance and associated factors in Swiss diabetes care – A cross-sectional study
Source: PLoS One. 2020 May 5;15(5):e0232686. doi: 10.1371/journal.pone.0232686 (PMC7200167; doi:10.1371/journal.pone.0232686)
Supplement: Attachment — Submitted filename: Response to Reviewers.docx [file pone.0232686.s012.docx]

| **Reviewer and item nr.** | **Reviewer / Editor comment** | **Author response** |
| --- | --- | --- |
| 1.1 | Page 7, lines 134–136: The cumulative QI performance ranged from 0 to 6. Please clarify the use of the hierarchical multivariable “binomial” regression model for an ordinal outcome. Did you mean a hierarchical multivariable ordinal logistic regression model where the odds would be interpreted as the odds of having more QI performance? | Thank you for your comment. By binomial regression, we meant that the dependent variable was defined as the – binomially distributed - cumulative QI performance: (cumulative QI performance, 6- cumulative QI performance), where the odds would be interpreted as the odds of satisfying an individual QI. We added more details to the manuscript (lines 139 – 140 in the manuscript with tracked changes) and hope that it is now better understandable what we meant. |
| 2.1 | In my previous review I suggested clarifying what you mean by “Structural data on participating GP are collected at individual project entry.” (line 75). Thank you for adding further detail to explain that this was done manually. However, I feel you should tell the reader who collected this information - I presume the research team? | Thank you for your comment. We clarified further, as you suggested, that the GPs self-reported the structural information to our study nurse (lines 78 - 80 in the manuscript with tracked changes) |
| 2.2 | There are still some typos in places throughout the manuscript so would advise careful proofreading before submission e.g. However, one can indirectly conclude from medication prescriptions that, the majority of included patients were patients with type 2 diabetes. | Thank you for pointing out this specific sentence. We made corrections to this sentence and carefully proofread the whole manuscript and made some minor changes throughout. |
| 2.3 | In the previous review I suggested rephrasing this sentence (line 188) as it as unclear what was meant. I suggest further rewording as this could be clearer and have included amendment:  “Number of years since diabetes diagnosis was much shorter in our study, which is explained by the fact limited data were available from before GPs participated in the FIRE project.” | Thank you for your suggestion and for clarifying its meaning. We changed the sentence as you suggested (lines 217 -219 in the manuscript with tracked changes). |
| 2.4 | In my previous review I noted that the Stone et al study (line 195) was missing a reference and that I thought this sentence was meant to be connected to the previous line. Thank you for adding the reference to the study. It is always helpful for the reader to have the reference cited a that place in the text even if the article was introduced earlier in the manuscript, particularly as you are using Vancouver referencing style. You did not address the second part of this comment. The sentence begins with ‘whereas’ and so hence my question about whether it should be connected to the preceding sentence. For example, I suggest it would read as:  "The proportions of patients fulfilling the process and outcome indicators for BP and HbA1c were highly comparable to the methodological similar study of van Doorn-Klomberg et al. 2015 [13], whereas the European cross-sectional study by Stone et al. 2013 [27] reported process indicators above 90%." | Thank you for your suggestion. We adapted the sentence as you suggested (line 224 in the manuscript with tracked changes). |
| 2.5 | Thank you for amending line 224. However, this sentence is still unclear. I think it should read as follows such that you clarify the direction of the effect:  "This finding is not completely in line with Lustman et al. 2016 [37], reporting a positive effect of continuity of care on the major clinical outcomes | Thank you for your comment. We changed the sentence as you suggested and clarified the direction of the effect (line 253 in the manuscript with tracked changes). |
