# Supplementary material for: Quality performance and associated factors in Swiss diabetes care – A cross-sectional study
Source: PLoS One. 2020 May 5;15(5):e0232686. doi: 10.1371/journal.pone.0232686 (PMC7200167; doi:10.1371/journal.pone.0232686)
Supplement: Attachment — Submitted filename: Reviewer comments - PONE-D-19-32658 .pdf [file pone.0232686.s010.pdf]

Review comments to the author

I found this manuscript interesting. However, I think you could make it far more relevant by revisiting the justification and implications/conclusions. I have included some specific comments below. In general, there are many typos and grammatical errors throughout. I have highlighted several instances but I may have missed some and so I would advise carefully proofreading subsequent versions of this manuscript.

<u>Abstract</u>

(line 33) typo: *We assessed the association of individual QI ~~performance of~~(i.e., process and intermediate outcomes: ~~for~~ glycated hemoglobin, blood pressure and cholesterol) ~~and associations of individual~~ and cumulative QI ~~performance~~ with GP and patient characteristics.*

(line 25) "*In Switzerland, no data on the pay-for-performance approach exists and the role of QIs has been marginal.*" It is unclear here what you mean by the 'role of QIs'. Do you mean that introducing and monitoring indicators has not led to an improvement in care?

In general, I would suggest use the term 'patients with diabetes' rather than 'diabetes patients' where possible throughout the manuscript.

(line 42) typo: *number of consultations (OR: 1.020, CI: 1.016-1.023)* – keep the number of decimal places used for OR consistent

I appreciate the wordcount here but would be good, if possible, to give the reader some sense of how the different data were collected i.e., at this point when reading about the study I do not know whether the performance indicators are based on extraction from medical records as part of an audit, or another study, and whether the details about the practice and the GPs were collected from a different source.

Introduction

(line 49) typo: "*The use of quality indicators (QI) and contingent incentives aim to improve process and outcomes in clinical practice*"

The following paper may also be useful background:

- Huang, J., Yin, S., Lin, Y., Jiang, Q., He, Y. and Du, L. (2013), Impact of pay-for-performance on management of diabetes: a systematic review. JOURNAL OF EVIDENCE-BASED MEDICINE, 6: 173-184. doi:10.1111/jebm.12052

(line 61) You state that: *"Currently, a randomized controlled trial (RCT) testing the P4P approach in Swiss primary care using clinical routine data is ongoing (15). The data of this trial offer the opportunity to study the characteristics and QI performance of the P4P study population and to analyze associations of QI performance with practice, GP and patient covariates."* While I agree this provides justification for the trial, it does not clearly provide justification for the current paper i.e., while it is important to examine the association of different factors (patient, GP, practice level etc) and quality performance. I would suggest including a line here to justify the current study e.g. did it inform the decision to conduct the trial or will it inform the trial and if so, how? I see that in the Methods you mention that data collection for the current study was conducted as part of the trial – it may be worth clarifying this earlier i.e., that the trial was progressing regardless but that it provided an opportunity to explore this association between patient, GP, practice factors and QI.

(line 57) Again, relating to study justification, you state "*From literature, we know that not only GP, but also patient characteristics influence QI performance (7-10)*". Since the association with *practice* factors is also mentioned later, should this be introduced earlier?

Methods

Since you mention the introduction of P4P in Switzerland and how the current study uses data from a trial of P4P, I think it would be important to provide some more detail here about the P4P programme e.g. what it involves and when it was introduced. When reading this section it is not immediately clear whether the data you use comes from baseline *before* P4P was introduced or the control group, or whether you are using all data returned to the database from 2017 to 2019 during which P4P was in place. To help the reader it may be useful to provide a figure or timeline, showing

data collection points etc. Figure 1 shows the flowchart and I think looking at this that you used data from the retrospective 12-month period and not the intervention group. If the latter is the P4P group then this suggests your data comes from practices and patients who are not within a P4P system - this should be made clearer.

Some acronyms might be unfamiliar to readers (e.g. ICPC, EMR), therefore would suggest spelling these out where possible.

(line 75) I would clarify what you mean by "*Structural data on participating GP are collected at individual project entry*." – does this mean GP/nurses/other staff entered this additional data to the EMR?

Your use of dates should be consistent - month and years are used in places but "01.01.2017" also used.

(line 86) In the 'Participants' section you report "*71 GPs from 43 different practices were willing to participate*". I feel this should be a result. I see you have reported this information again at the start of the 'Results' section.

Maybe I missed it, but you do not appear to state whether these are patients with type 1 or type 2 diabetes or both types are included in your sample. On this point, did you consider conducting the analysis separately by type or adjusting for this variable in your regression models?

In terms of the statistical analysis, were any further covariates included in the models or just those listed? I would suggest a footnote on the table to clarify these are adjusted OR and what variables they were adjusted for.

You should include an explanation of how cumulative QI performance was calculated - do you mean a composite QI here? Was there a scoring system?

I know it is mentioned in the introduction, but in connection with the previous comment I would suggest listing which specific QI were used for the current study in the Methods section. You mention the type of data collected but are not explicit about which data corresponds to the QI to examined.

(line 110) You state one objective is "*To examine the association of QI performance with practice, GP and patient covariates"* (see typo here also*),* however in the analysis section practice factors are described and listed under 'GP characteristics'. Therefore, this leads me to think that these factors are being explored at the GP level – I would suggest you keep the terminology consistent throughout the paper, so either describe these as three (patient, GP, practice) or two (patient, GP) separate variable categories or levels and be clear about what comes under each.

(line 120) typo: "*Number of medications and BMI were not considered as covariates for the regression model as they were used to identify certain comorbidities*"

Results

If space allows I think it may be helpful to have a descriptive Table 1 showing the profile of GPs and/or patients. For example, much of the text about the different patient medications and co-morbidities could be captured in a table rather than listing in the text.

It is good practice to provide the % and the N – if you have your descriptive table(s) as suggested then you can refer the reader here for the numbers.

(line 132) typo: "*These patients were ~~in~~ 57% male and had a mean age of 68.3 years (SD 13.4)."*

(line 137) typo: *"On average, patients had eight consultations (IQR: 5-15) at the GPs' practice (or 'a GP practice') in the 12 months preceding baseline."*

In the text it is not always clear whether you mean targets achieved or processes performed. These are different and should be made explicit when reporting the results. For example, in line 161 you state *"higher age was significantly associated with a higher HbA1c QI performance but with a lower cholesterol QI performance"* . Should 'QI performance' in this case be taken to mean both measurement of HbA1c (process) and achievement of <7.5% (target achieved)?

In Table 1 you show the % patients achieving QI. There are BP and HbA1c targets mentioned (i.e. <140/85mmHg, <7.5%, < 5 mmol/l). These should have been mentioned in the <u>Methods</u> when explaining the QI to be examined. I realise these are common targets in diabetes management, but I

feel it would be important to provide a rationale for using them i.e., are they used in national and/or international guidelines. Later in the manuscript you mention the American Diabetes Association guidelines. If the targets you use are based on these guidelines I would suggest citing them.

(line 146) "*93.8% of the patients had at least one comorbidity, whereas 37.9% of the patients had three or more comorbidities*." – do not begin sentences with a numeral. Please amend this in the rest of the manuscript.

In the section titled 'Associations with QI performance' I would report the OR and 95% CI in the text, particularly the significant findings, perhaps referring the reader to the table to review the non-significant findings.

I suggest rephrasing line 159 "*GP characteristics had almost no significant influence on QI performance*" as is this is potentially confusing. It would be better to say: 'Some GP characteristics were associated with QI performance'. I would also advise revising this expression at the start of the discussion.

Similarly, I think saying 'lower' and 'higher' and 'lowered' QI performance (lines 158 -166) is potentially confusing as this implies a continuous rather than binary outcome. You are examining the association of these factors with the odds of either performing a process or achieving a target, so I would reword the text throughout this section to make this clear.

(line 168) 'Cumulative QI' again is mentioned but it is unclear what is meant here – looking at this table it seems the individual QI are again reported rather than a summary or composite.

<u>Discussion</u>

(line 182) "*In this study, we explored associations of QI performance with patient, GP and practice characteristics."* – I would rephrase this line, as earlier in the manuscript you lead with the exposure (i.e. GP, practice, patient factors) and finish with the outcome (i.e. QI performance) and this is the convention. In general, I would check this phrasing throughout the manuscript to ensure consistency - I believe the order may be reversed in the results also.

I suggest rephrasing this sentence (line 188) "*Number of years since diabetes diagnosis was much shorter in our study, which is explicable through limited data availability before GPs participated in the FIRE project.*" as it is unclear what you mean – is it the case that people may have been diagnosed earlier but you do not have access to data on these people?

(line 189) "Regarding clinical outcomes, our study population achieved slightly better than the other two Swiss populations." – I think there may be a word missing here? Slightly better ____?

You refer to other studies and how it is difficult to compare their findings your study. I think you need to reflect further on the existing literature and provide a little more detail. For example, you mention (line 193) "*Our results for process and outcome indicators for BP and HbA1c were highly comparable to the methodological similar study of van Doorn-Klomberg et al. 2015*" - I assume you mean the proportions in terms of targets met or processes performed, but some further explanation would help.

The Stone et al study (line 195) is missing a reference. I think this sentence was meant to be connected to the previous line.

I would not use the phrase "well above 90%" (line 196). Either provide a range or simply state they were over 90%. In general, it is better to give readers a sense of the scale i.e. %. This also applies to the next line where you comment that the other Swiss study found "slightly higher" rates.

(line 200) You state that "*Reasons for such poor performances might be clinical inertia to intensify treatment, poor patient adherence or misjudgment by our QI scheme (25, 26).*" – I would be interested to know whether you think there is something about the patient or GP population studied which might explain why issues such as inertia would be more common, than, for example, in the Djalali et al. 2014 study which you say is the same EMR database. Does this imply these are similar patients and GPs?

On this point, you mention in the strengths and limitations, using the "*full spectrum*" (line 236) of patients and that "*could show high generalizability of our data due to given patient characteristics.*" – I feel the descriptive Table 1 in the Results showing the patient profile would be helpful. In the text you only describe patient in terms of age, sex and diabetes duration, therefore the reader may not understand how this compares to the general population with diabetes.

It is not clear what you mean by 'misjudgement by our QI scheme' (line 200) so this needs to be explained.

You may want to consider what other (unmeasured) factors may influence achievement of QI. For example, is patient socio-economic status something which could have been included in the analysis – I see that you mention this under the limitations section, but you do not expand on why this is relevant to your broader findings. If you feel patient adherence is an issue, then number of medications would have been an interesting variable to examine, though I know this was used to identify comorbidities. The following review may be useful to consult:

- Hertroijs DFL, Elissen AMJ, Brouwers MCGJ, et al. Relevant patient characteristics for guiding tailored integrated diabetes primary care: a systematic review. *Primary Health Care Research & Development*. 2018:1-24.

You might consider commenting further on the finding that increased patient age and longer duration were both associated with poorer performance on HbA1c QI, while not associated with BP and cholesterol. For example, what are the implications? Is this something other studies have found? Perhaps this could be linked with the limitation you mention regarding having approximated duration by using the first record as the onset of diabetes.

(line 205) typo: "*Regression analysis revealed that included characteristics had ~~no to~~ only very small effect on process and outcome indicators.*"

(line 206) What is meant here?: "*Most significant effects were found on patient level, and the largest effect was an increasing number of diabetes-relevant comorbidities*." The largest effect on what? What this a positive effect? By 'most significant effects' do you mean that more patient level factors were associated with QI performance?

Lustman et al. 2016 is missing a citation

(line 224) "*Which is contradictory ~~ing~~ to Lustman et al. 2016 reporting an effect of continuity of care on the major clinical outcomes.*" What is meant here? They found continuity of care had a negative effect on outcomes? How did they define continuity of care in their study?

(line 227) typo: "*However, our model also revealed that ~~unexplained~~ variation exists between practices and GPs, which cannot be explained by our model*"

Where you mention other unmeasured GP factors such as culture, working style etc., it may be worth citing some qualitative work which has been done in the area of barriers and facilitators of diabetes management in primary care. Some examples below:

- Rushforth B, McCrorie C, Glidewell L, et al. Barriers to effective management of type 2 diabetes in primary care: qualitative systematic review. *Br J Gen Pract*. 2016;66(643):e114-27.
- Zhang JQ, Van Leuven KA, Neidlinger SH. (2012) System Barriers Associated With Diabetes Management in Primary Care. *The Journal for Nurse Practitioners* 8(10):822-7.
- Nam S, Chesla C, Stotts NA*, et al.* (2011) Barriers to diabetes management: patient and provider factors. *Diabetes Res Clin Pract* 93(1):1-9.

I would advise not using the term 'diabetic patients'; instead 'people with diabetes' or 'patients with diabetes'. See https://care.diabetesjournals.org/content/40/12/1790 and the discourse around the use of terminology.

In the strengths and limits you mention 'potential missing data' (line 244). It would be helpful report in the Results whether any of the variables you used had missing data. As you only report % in the text it is difficult for the reader to determine the level of missingness.

The meaning of this sentence (line 250) is unclear and needs to be rewritten "*This bias is probably of larger issue with lower performance rates as in QI for cholesterol."*

(line 252) You mention *"We tried to minimize the amount of missing data by setting a minimum standard of data availability for each GP to be contacted for recruitment*." . This is important and should be mentioned earlier in the Methods section when explaining participants. From the way you describe it in results "*125 GPs met the data availability criteria of a) delivering data on a regular*

basis since 01.01.2017" it was not clear to me that there was a threshold, or what is meant by 'regular basis' – do you mean they had to deliver a complete dataset annually or at some other interval?

I think there is potential to expand on your overall implications and conclusions and make these stronger and better situate them in the broader literature. For example, given there were a number of covariates which you could not explore in your analysis: 1) do you feel there is scope for future work (and what would this look like?), 2) is there scope to expand on (e.g. to include further variables) or improve (e.g. your comment about missingness) the type of data collected in the FIRE database, 3) do you think this database should or can be used for monitoring quality of care and providing feedback to GPs/practices in the future?

As I commented earlier there needs to be a more explicit justification for the current study in the Introduction. Once this is made clearer it will be easier for you to link your conclusions and implications back to the reason for conducting the study. For example, considering your conclusion regarding the 'room for improvement', was your initial aim to determine whether the quality of Swiss diabetes management *needs to* be improved and *how to* do so and/or *identify* factors to inform case-mix adjustments? How do your findings inform the P4P trial? Earlier in the Introduction you mention the lack of understanding around QI performance within P4P systems, particularly in Switzerland. However, in the Discussion there is no mention of P4P and how your findings relate to research from other countries which have introduced P4P – for example, the Quality and Outcomes Framework in the UK. I understand this omission if you used data from the patients and GPs who were not in the P4P intervention group. However, even if you looked at QI performance in a group who are not within P4P system, still I think you need to be clearer about the purpose and relevance of this current study, how it relates to the trial and how it fits in with the bigger picture.
